# Correction: Network Models Predict that Reduced Excitatory Fluctuations Can Give Rise to Hippocampal Network Hyper-Excitability in MeCP2-Null Mice

**DOI:** 10.1371/journal.pone.0103906

**Published:** 2014-07-23

**Authors:** The PLOS ONE Staff

## Notice of Republication

This article was republished on July 7, 2014, to correct several errors that were introduced during the typesetting process. Please download this article again to view the correct version. The originally published, uncorrected article and the republished, corrected article are provided here for reference.

## Supporting Information

File S1 Originally published, uncorrected article.
(PDF).

File S2 Republished corrected article.
(PDF).
