# Supplementary material for: Correction: Network Models Predict that Reduced Excitatory Fluctuations Can Give Rise to Hippocampal Network Hyper-Excitability in MeCP2-Null Mice
Source: PLoS One. 2014 Jul 23;9(7):e103906. doi: 10.1371/journal.pone.0103906 (PMC4108399; doi:10.1371/journal.pone.0103906)
Supplement: File S1 — Originally published, uncorrected article. (PDF). [file pone.0103906.s001.pdf]

OPEN ACCESS Freely available online

PLOS | ONE

Network Models Predict that Reduced Excitatory Fluctuations Can Give Rise to Hippocampal Network Hyper-Excitability in MeCP2-Null Mice

Ernest C. Y. Ho[1,2¤], James H. Eubanks[1,3], Liang Zhang[1,4¶], Frances K. Skinner[1,2,4*¶]

1 Toronto Western Research Institute, University Health Network, Toronto, Ontario, Canada, 2 Department of Physiology, University of Toronto, Toronto, Ontario, Canada, 3 Department of Surgery (Neurosurgery), University of Toronto, Toronto, Ontario, Canada, 4 Department of Medicine (Neurology), University of Toronto, Toronto, Ontario, Canada

Abstract

Rett syndrome is a severe pediatric neurological disorder caused by loss of function mutations within the gene encoding methyl CpG-binding protein 2 (MeCP2). Although MeCP2 is expressed near ubiquitously, the primary pathophysiology of Rett syndrome stems from impairments of nervous system function. One alteration within different regions of the MeCP2-deficient brain is the presence of hyper-excitable network responses. In the hippocampus, such responses exist despite there being an overall decrease in spontaneous excitatory drive within the network. In this study, we generated and used mathematical, neuronal network models to resolve this apparent paradox. We did this by taking advantage of previous mathematical modelling insights that indicated that decreased excitatory fluctuations, but not mean excitatory drive, more critically explain observed changes in hippocampal network oscillations from MeCP2-null mouse slices. Importantly, reduced excitatory fluctuations could also bring about hyper-excitable responses in our network models. Therefore, these results indicate that diminished excitatory fluctuations may be responsible for the hyper-excitable state of MeCP2-deficient hippocampal circuitry.

Citation: Ho ECY, Eubanks JH, Zhang L, Skinner FK (2014) Network Models Predict that Reduced Excitatory Fluctuations Can Give Rise to Hippocampal Network Hyper-Excitability in MeCP2-Null Mice. PLoS ONE 9(3): e91148. doi:10.1371/journal.pone.0091148

Editor: William W. Lytton, SUNY Downstate MC, United States of America

Received December 20, 2013; **Accepted** February 7, 2014; **Published** March 18, 2014

Copyright: © 2014 Ho et al. This is an open-access article distributed under the terms of the Creative Commons Attribution License, which permits unrestricted use, distribution, and reproduction in any medium, provided the original author and source are credited.

Funding: Funders: Natural Sciences and Engineering Research Council of Canada (NSERC) Discovery Grant (FKS, LZ); Canadian Institutes of Health Research (CIHR) MOP-106481 (JHE, LZ), and Ontario Brain Institute (LZ). Computations were performed on the GPC supercomputer at the SciNet HPC Consortium. SciNet is funded by: the Canada Foundation for Innovation under the auspices of Compute Canada; the Government of Ontario; Ontario Research Fund - Research Excellence; and the University of Toronto. The funders had no role in study design, data collection and analysis, decision to publish, or preparation of the manuscript.

Competing Interests: The authors have declared that no competing interests exist.

* E-mail: frances.skinner@utoronto.ca

¶ These authors are co-senior authors on this work.

¤ Current address: Department of Neuroscience, Brown University, Providence, Rhode Island, United States of America

Introduction

Rett syndrome is an X-linked genetic disorder that largely affects females, and stems from mutations in the gene encoding methyl-CpG-binding protein 2 (MeCP2) [1]. While the severity of symptoms differs between individuals [2–4], common impairments in Rett syndrome patients include loss of fine motor skills, failure to develop speech, impaired locomotive ability, breathing irregularities, diminished cognitive ability, and seizures [2,5,6]. Although MeCP2 is near ubiquitously expressed, the CNS is more affected than peripheral tissues as a consequence of impaired MeCP2 function. Electroencephalography (EEG) studies conducted on Rett syndrome patients illustrate altered neural function. Diminished brain rhythmic activity, slower evoked sensory responsiveness [7], and spontaneous epileptiform discharges are commonly observed [7–9]. To date, however, the underlying mechanisms that cause these alterations in network activity remain largely unknown.

To facilitate *in vivo* investigations, mouse models of Rett syndrome have been developed that recapitulate many of the cardinal features seen in Rett syndrome patients [10]. Several lines of experimental work in different MeCP2-deficient mouse models confirm that the absence of MeCP2 alters normal synaptic activity, although the effects are not identical between different brain regions. For example, decreased spontaneous excitatory activity has been observed within juvenile and adult MeCP2-deficient cortex and hippocampus [11–15], while heightened excitatory activity has been reported in brain stem and mid-brain loci [16–20]. Collectively, these data argue for the presence of microcircuit-specific changes in the MeCP2-deficient brain that can specifically influence the phenotype of larger neural networks, and ultimately contribute to impairments in behavioural performance, albeit in circuit or microcircuit specific manners.

One step towards obtaining a better understanding of how network dynamics influence Rett syndrome phenotypes is to use a 'reduced microcircuit' strategy to identify how synaptic alterations in a defined structure affect its network activities. The hippocampal formation is one neural structure that has commonly been the focus of such investigations. Previously, Zhang and colleagues [21] demonstrated the presence of robust, spontaneous, inhibitory-based slow frequency population activities in thick slice preparations from the adult hippocampus. Using data from these preparations, we created network models that were able to predict

how these slow population activities could be generated [22]. These mathematical models allow us to examine potential mechanisms responsible for network activity profiles seen in both normal and pathological hippocampal microcircuits. Using these same slice preparations as in [21], Zhang et al. [13] examined their population activities in the MeCP2-null mouse model. This study identified a seemingly paradoxical state: its intrinsic network activity is hyper-excitable [13,23,24], but its local spontaneous post-synaptic excitatory drive is diminished from that of wild-type [13]. These experimental results, together with understandings derived from our previous mathematical models, present an opportunity for us to gain insight into critical aspects occurring in Rett network dynamics. In this report, we exploit this to illustrate network model outcomes with strong phenotypic similarities to the network activity observed in the hippocampus of MeCP2-null mice. As such, we are able to identify a potential mechanism through which reduced excitatory fluctuations can give rise to reduced population activities, yet still promote hyper-excitable network responses.

Materials and Methods

Experiments

Animal subjects. All animal experimentation was conducted in accordance with the guidelines of the Canadian Council of Animal Care, and thoroughly reviewed and approved before implementation by the Toronto General and Western animal care committee (Protocols 882.6 and 1321.9). All surgeries were performed under general anesthesia, and every effort was made to minimize pain in the experimental subjects. MeCP2$^{tm1.1Bird}$ mice [25] and wild-type mice were obtained from The Jackson Laboratory (Bar Harbor, Maine) and maintained on a pure C57Bl/6 background. MeCP2$^{tm1.1Bird}$ mice were sacrificed between 65–85 days at age for *in vitro* electrophysiological assays. None of the mutant animals displayed complete immobility or a moribund appearance at the time of sacrifice, although each subject did display impairments in hind limb elevation reflex indicating the presence of Rett-like symptoms [26,27].

Brain slice preparation. Hippocampal slices (thickness 0.5 mm) were prepared as described previously [13,21,28]. In brief, mice were anaesthetized by an intra-peritoneal injection of sodium pentobarbital (70 mg/kg, Somnotol, WTC Pharmaceuticals, Cambridge, Ontario, Canada), and transcardially perfused with cold artificial cerebrospinal fluid (ACSF). The mice were rapidly decapitated, and their brains extracted and immersed in ice-cold oxygenated ACSF for 5 minutes before slicing. Hippocampal slices were then stabilized in an oxygenated (95% $O_2 - 5\%$ CO_2) ACSF at $32-33^{\circ}$C. for 1–6 hours before electrophysiological assessments. For the recordings, individual slices were held in a submerged chamber that was continually perfused with the oxygenated ACSF at $32-33^{\circ}$ C [13,29]. The ACSF used for this study consisted of (in mM): NaCl 125, KCl 35, NaH_2PO_4 1.25, $NaHCO_3$ 25, $CaCl_2$ 2, $MgSO_4$ 1.3 and glucose 10 (pH of 7.4 when aerated with 95% $O_2 - 5\%$ CO_2).

Electrophysiological recordings. Extracellular recordings were conducted using a dual channel amplifier (700A, Axon Instruments, Foster City, CA, USA) as described previously [13]. All electrophysiological data were acquired, stored, and analyzed using PCLAMP software (version 9, Axon Instruments). Extracellular recording electrodes were made with glass pipettes filled with a solution containing 200 mM NaCl and 5 mM HEPES (pH adjusted to 7.4). The extracellular recording electrode was placed in the CA3 subfield, largely in the somatic or cell body layer. The hippocampal slow population activities (hippocampal SPAs) assessed in this study were previously referred to as spontaneous rhythmic field potentials (SRFPs) in [13] and [21], and were also referred to as basal sharp waves (bSPWs) in [30], to distinguish them from SPWs that had an intermittent occurrence in association with strong excitatory activities in individual pyramidal neurons [13,30]. Afferent stimulations were done using a bipolar tungsten wire electrode (diameter 50 μm) and a Grass stimulator (S88) as described [13]. High frequency stimulation (HFS) was applied to the CA3 oriens area by repetitive stimuli at 80 Hz for 1 second (constant current pulses of 0.1 ms duration and 100–150 μA).

Simulations

Model Description and Parameter Choices. The basic set of differential equations governing both the model inhibitory interneurons and pyramidal cells [31,32] is (we use the letters l,m to denote general cell number indexing).

$$\begin{aligned} V^l &= V_{reset} \text{ (when } V^l \text{ rosses } V_{max} \text{ from below)} \\ C \times \frac{dV^l}{dt} &= I^l_{int}(t) + I^l_{syn}(t) \text{ (otherwise)} \end{aligned} \quad (1)$$

where I^l_{int} represents the intrinsic current of the neuron in question. The specific capacitance C is assumed to be 1 $\mu F/cm^2$.

I^l_{int} has the following form.

$$\begin{aligned} I^l_{int} &= \alpha \times (V^l - V_{rest}) \times (V^l - V_{th}) - n_l + I_{ext} \\ n_l &\to k \times n_l + d \quad \text{(when } V^l \text{ crosses } V_{peak} \text{ from below)} \\ \frac{dn_l}{dt} &= \lambda \times (\beta \times (V^l - V_{rest}) - n_l) \quad \text{(otherwise)} \end{aligned} \quad (2)$$

Equation set 2 is the most general mathematical representation of the firing of neurons with spike frequency adaptation. In the above equation set, the parameters V_{rest} and V_{th} represent the reseting membrane potential value and the threshold membrane potential value respectively (in mV). The variable n is an auxiliary current which represents the spike frequency adaptation characteristics of the neuron, while the parameters λ and β control the magnitude of this adaptation. The parameter α partially controls the f-I characteristics of the neuron close to the threshold.

Excitatory, pyramidal cell model: We use equation set 2 and the models are based on those determined in [32] with spike frequency adaptation characteristics of CA3 pyramidal neurons, and using a surface area of 25000 μm^2.

Inhibitory, interneuron cell model: We use a reduced version of equation set 2 for inhibitory interneurons since there is minimal spike frequency adaptation and narrow spike width, and also, to be consistent with models used in our previous work [22]. In particular, we set $V_{rest} = V_{th}$, $\beta = 0$ and $k = 0$ whenever we use equation set 2 to represent inhibitory interneurons. The reduction is equivalent to the mathematical representation of inhibitory interneurons used in [22] and [33] (with $p = 2$ in their model).

Synaptic model: The synaptic current I^l_{syn} is represented as,

$$\begin{aligned} I^l_{syn} &= \sum_{m;m \neq l} \Gamma_{m \to l} \times g^{m \to l}_{syn} \times s_m \times (E_{\{e,i\}} - V^l) \\ s_l &\to s_l + s_{max} \text{ (when } V^l \text{ crosses 0 from below)} \\ \frac{ds_l}{dt} &= -\frac{s_l}{\tau_{\{e,i\}}} \text{ (otherwise)} \end{aligned} \quad (3)$$

where $E_{\{e,i\}}$ is the excitatory or inhibitory reversal potential value

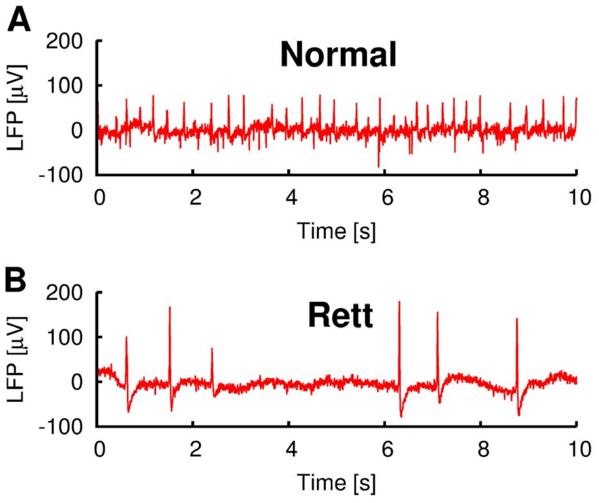

Figure 1. Slow population activities in experiment for wild type and MeCP2-null mouse hippocampus. The traces are offset so that their baselines are around 0 μV. (**A**) Wild type. (**B**) MeCP2-null.
doi:10.1371/journal.pone.0091148.g001

respectively (whether the reversal potential is excitatory or inhibitory is dependent on the particular neuron–that is neuron m–from which neuron l is connected). s_l represents the synaptic gating variable of neuron l. For computational efficiency and as done before [22], we use a discontinuous model to represent the synaptic activation (equation set 3) whenever there is a spike (comparative simulations indicate that results are similar to those using a continuous model). Parameters $\tau_{\{e,i\}}$ and s_{max} (for s_l) denote respectively the excitatory (inhibitory) synaptic decay time constant and the value of the maximal opening of the synaptic gates per spike (whether the decay time constant should be excitatory or inhibitory is dependent on whether neuron l itself is a pyramidal cell or an inhibitory interneuron). $\Gamma_{m\to l}$ is the connectivity matrix element being either 1 (connected) or 0 (not connected) depending on whether neuron l receives synaptic input from neuron m.

Population model representation - local field potential (LFP) analogue: In our simulations, we represent the experimentally observed LFP modulations due to SPAs (as seen in Figures 1A and B) by the average value of inhibitory, synaptic gating variables. In other words, we define our LFP analogue as $\frac{\sum_{m;m\in inh} s_m}{N_{inh}}$, where N_{inh} is the number of model inhibitory interneurons in the simulated network. This definition is identical to the definition of LFP analogue as we used before [22]. Since LFPs have been shown to be clearly correlated with firing of inhibitory cells and less so with excitatory cells, we only use inhibitory, synaptic gating variables in generating our LFP analogue from (inhibitory, inhibitory-excitatory) model networks.

Network connectivities and external driving forces. We use different network configurations and connectivities for various figures to illustrate concepts and results. All these different network configurations are built upon mathematical representations of inhibitory and/or excitatory neurons (equation sets 1 and 2), with the neurons being connected by synapses (equation set 3). As assumed before [22], since the population activities seen in experiment do not seem to preferentially involve specific inhibitory cell types, our inhibitory models focus on fast-spiking cell types since they constitute the majority of inhibitory cells. However, the connectivity matrix elements $\Gamma_{m\to l}$ are not the same for the different network configurations. In setting up $\Gamma_{m\to l}$ for each simulation, we assign each matrix element randomly (with either 1 or 0) with the probability of getting 1 being P (see Table 1). As well, the format of the external current I_{ext} used to drive the neuronal populations is different for the various simulations. We now briefly outline the network configurations used for the different simulations in this work.

Inhibitory Networks and Virtual Excitatory Networks: To mimic a fluctuating background excitatory synaptic environment, each model inhibitory interneuron is driven by I_{ext} with the form [34].

$$
\begin{aligned}
I_{ext} &= g_e^l(t) \times (E_e - V^l) \\
\frac{dg_e^l(t)}{dt} &= -\frac{g_e^l(t) - g_{e0}}{\tau_e} + \sqrt{\frac{2\times\sigma_e^2}{\tau_e}} \times \chi_e^l(t)
\end{aligned}
\tag{4}
$$

where E_e is the excitatory reversal potential and τ_e is the excitatory time constant. The excitatory conductance g_e is a stochastic variable following the Ornstein-Uhlenbeck process (equation set 4) with mean g_{e0} and SD σ_e (excitatory fluctuations). χ_e is the term which underlies the stochasticity of g_e. The quantity $\int_0^{\in} \chi_e(s)ds$, $\in > 0$ can be represented as a Gaussian distribution with mean zero and variance $\in$. Further details on the numerical implementation of the stochastic elements of equation set 4 can be found in [22].

In Figure 2, we use a network configuration that consists of mathematical representations for inhibitory interneurons (equation sets 1–3) and "virtual" representations for the excitatory cell population (equation set 4). Each model inhibitory interneuron is connected with all the other inhibitory interneurons in the network, while receiving fluctuating input from the "virtual" excitatory cell population. This network configuration is identical to the one that was used in [22], except that here we use 100 model interneurons instead of 120 used there. We use all-to-all connectivity here noting that when non all-to-all connectivity was explored in [22], the determined mechanism being exploited here was maintained.

Inhibitory and Excitatory Networks: The effects of the "virtual" excitatory cell population can be realized by explicitly including model pyramidal cells. In Figure 3 we show the same simulations as in Figure 2 respectively, except that the model inhibitory interneurons are now driven by actual pyramidal cells rather than the virtual input as in equation set 4. We synaptically connect the model inhibitory interneurons with a population of 800 model pyramidal cells (so that the ratio of inhibitory to excitatory cells is approximately in line with the value in the CA3 area of the rat hippocampus). The firings of the pyramidal cells provide the input for inhibitory interneurons (while setting the I_{ext} to model inhibitory interneurons to zero). We drive each pyramidal cell with a constant I_{ext}. We ensure that the output from the pyramidal cell population is fluctuating by imposing heterogeneity of the I_{ext}'s given to different pyramidal cells. The connectivity between E-cell and I-cell populations is mainly based on obtaining appropriate fluctuating outputs seen experimentally.

Excitatory Networks: In Figure 4, we use a collection of 800 pyramidal cells to examine network bursting or sharp wave-like responses as observed experimentally. In particular, we show how the magnitude of excitatory fluctuations can affect network bursting. The excitatory connectivity of these 800 pyramidal cells is loosely based on CA3 excitatory model networks [35]. We vary the excitatory-excitatory conductance values ($g_{syn}^{e\to e}$) between different simulations, and we determine the threshold $g_{syn}^{e\to e}$ for which network bursting occurs. We introduce driving forces and

Table 1. Synaptic parameters used for network simulations.

Parameter	Description	Units
E_i	Inhibitory reversal potential	mV
E_e	Excitatory reversal potential	mV
τ_i	Inhibitory synaptic decay time constant	ms
τ_e	Excitatory synaptic decay time constant	ms
s_{max}	Maximal opening of inhibitory gating variable per spike	*dimensionless*
g_{e0}	Mean background excitatory drive to model neurons (see equation set 4)	mS/cm^2
σ_e	Background excitatory fluctuation level (see equation set 4)	mS/cm^2
$g_{syn}^{i\to i}$	Unitary conductance from an inhibitory interneuron to another interneuron	mS/cm^2
$g_{syn}^{e\to e}$	Unitary conductance from a pyramidal cell to another pyramidal cell	mS/cm^2
$g_{syn}^{e\to i}$	Unitary conductance from a pyramidal cell to another interneuron	mS/cm^2
$g_{syn}^{i\to e}$	Unitary conductance from an interneuron to another pyramidal cell	mS/cm^2
$\Gamma_{m\to n}$	Connectivity matrix element (with values of either 1 or 0) between neuron m and n	*dimensionless*
$P(i\to i)$	Connection probability from one interneuron to another interneuron	*dimensionless*
$P(e\to e)$	Connection probability from one pyramidal cell to another pyramidal cell	*dimensionless*
$P(e\to i)$	Connection probability from one pyramidal cell to another interneuron	*dimensionless*
$P(i\to e)$	Connection probability from one interneuron to another pyramidal cell	*dimensionless*

doi:10.1371/journal.pone.0091148.t001

basal excitatory fluctuations to these networks. In Figure 4, we inject a constant current I_{ext} to drive each cell. Background excitatory fluctuations are introduced via the heterogeneity of I_{ext} (as in the pyramidal cells of Figures 3). In other words, each cell receives a generally different constant I_{ext} than other cells. The amount of excitatory fluctuation levels can be controlled by the magnitude of I_{ext} heterogeneity.

Tables 1 and 2 list all the parameters we use for the simulations, and Table 3 is a detailed summary of the parameters and network connectivities for all the simulations. We perform most of the simulations on the GPC supercomputer at the SciNet HPC Consortium ([36], http://www.scinethpc.ca). We use the Euler algorithm for numerical integration. Integration time step for each simulation ranges from 0.01 to 0.02 ms.

Results

In the results here, we first illustrate and describe the experimental findings of [13], as well as reviewing our previous mechanistic findings [22]. We then directly show the implications of our mechanistic understanding using model networks that emulate Rett population activities observed by [13]. We use inhibitory model networks with virtual excitatory drive, similar to those used previously [22]. As well, we use expanded versions that directly include excitatory cells. Given these implications, we use additional excitatory network simulations to explain the apparent paradox regarding hyper-excitable responses in brain slices from MeCP2-null mice, despite reduced excitatory activities.

Mathematical model mechanism provides suggestions for observed changes of hippocampal population activities in slices from MeCP2-Null mice as compared to wildtype

Spontaneous population activities produced from thick slice preparations of hippocampus are illustrated in Figure 1. Specifically, Figures 1A and B depict extracellular local field potential (LFP) recordings of hippocampal slow population activities for the wild type and MeCP2-null mouse hippocampus respectively. These LFP population activities are inhibition-based (i.e. are due to the coherent firing of inhibitory interneurons, and consequently IPSPs on pyramidal cells), originate from the CA3 area of the mouse hippocampus, and are dependent on a balance of GABAergic and glutamatergic synapses [13,21,22,30]. Their frequencies can range from 0.5 to 4 Hz. Depending on the location of the extracellular probe, a single hippocampal population activity episode can have a magnitude up to 0.5 mV.

Comparing Figures 1A and B, one notices that although these population activities exist in both wild type (Figure 1A) and MeCP2-null (Figure 1B) mouse hippocampal slices, there are clear differences in their characteristics. Most prominently, there are much fewer "events" in the MeCP2-null mouse hippocampal slice (Figure 1B) as compared to the wild type ones (each figure depicts a 10-second stretch of reading). As a result, the population frequency in the MeCP2-null sample is much lower, approximately 0.5 Hz as opposed to the wild type sample of about 3.5 Hz in this illustration (frequencies of hippocampal population activities from wild type samples can be between 1 Hz and about 4 Hz–see Figure 1C of [13]). Also, the slow population activity amplitudes in the MeCP2-null sample are somewhat higher than in the wild type sample.

What mechanism(s) could cause the observed changes in these hippocampal population activities in MeCP2-null samples? Zhang et al. [13] compared the electrophysiological properties of the intracellular correlates, and found no significant difference between wild type and MeCP2-null samples. As such, it is likely that the cause is in the synaptic component of the network, as has been suggested by other studies [11]. Previously, [22] developed a mathematical model which described the generation of inhibitory-based slow population activities, which we termed SPAs. Subsequent model analyses revealed that SPAs are the result of network multistability, which in turn is due to the interactions between synaptic and intrinsic properties of interneurons. Excitatory fluctuations were key since they play the role of driving the network between different stable states to bring about the existence

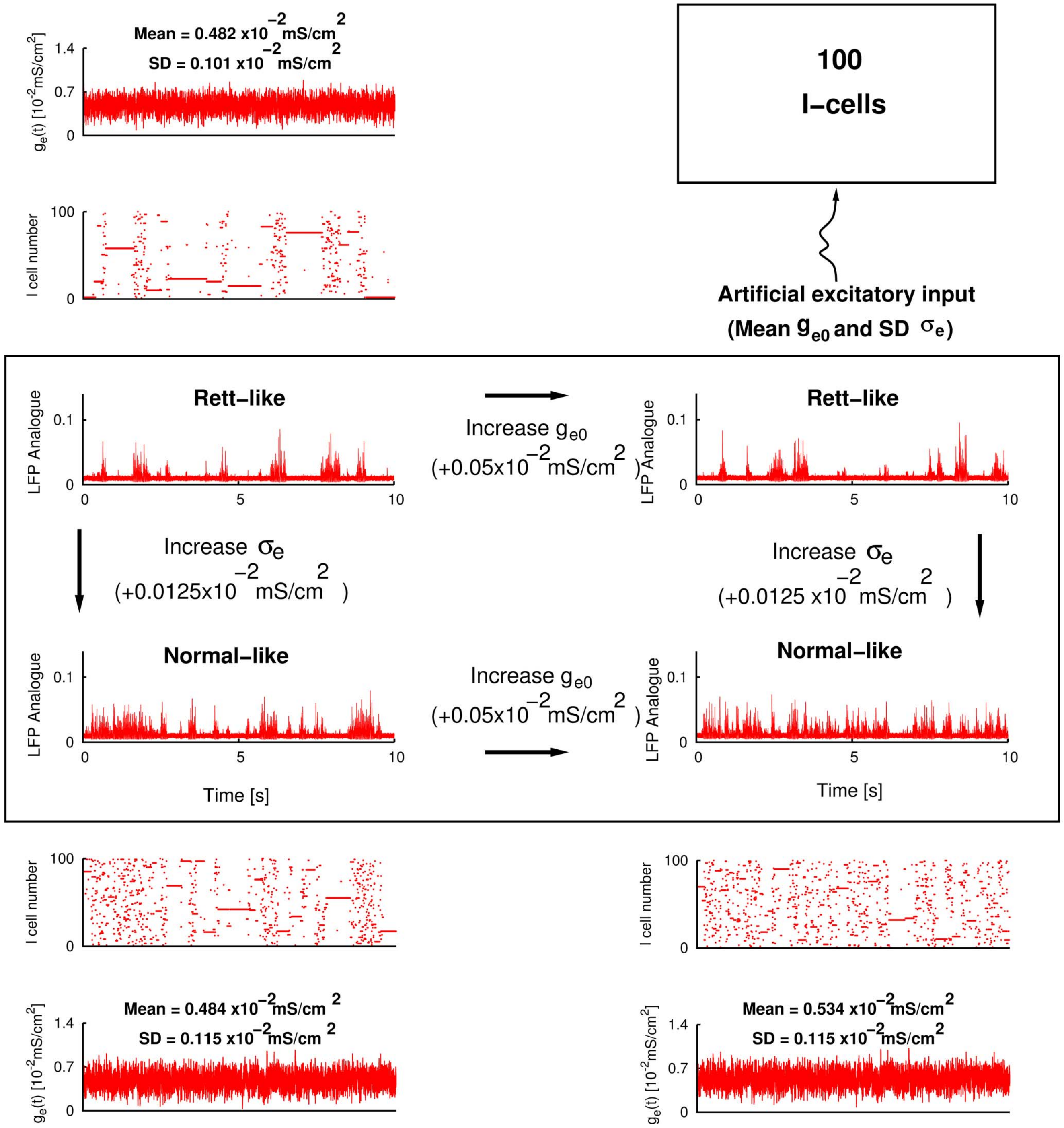

Figure 2. Simulated hippocampal slow population activities for normal and Rett. Box in the middle shows Rett and normal-like LFPs. Above and below the box are raster plots of the inhibitory cells and fluctuating excitatory input received by inhibitory cells for the particular example shown. Also shown is a schematic of the model setup. **Top left** $g_{e0}=0.00483$, $\sigma_e=0.00102$. **Top right** $g_{e0}=0.00533$, $\sigma_e=0.00102$. **Bottom left** $g_{e0}=0.00483$, $\sigma_e=0.001145$. **Bottom right** $g_{e0}=0.00533$, $\sigma_e=0.001145$.
doi:10.1371/journal.pone.0091148.g002

of SPAs. Given this, one of the predictions from this simulation work is that SPA characteristics are more dependent on the amount of excitatory fluctuations, but less so on mean excitatory levels. In particular, our previous work predicts that a decrease in the amount of excitatory fluctuations would lead to increased SPA amplitudes but decreased SPA frequencies. Zhang et al. [13] demonstrated that excitatory activities are decreased in MeCP2-null samples (Figure 6 of their publication). Furthermore, attenuating glutamatergic activities by NBQX or ADAC (an A1 receptor agonist) perfusion in wild type mouse hippocampal slices reduces frequencies of SPAs occurring there (blanket reduction in excitatory glutamatergic activity almost always leads to smaller excitatory fluctuation levels). Taken together, it may be

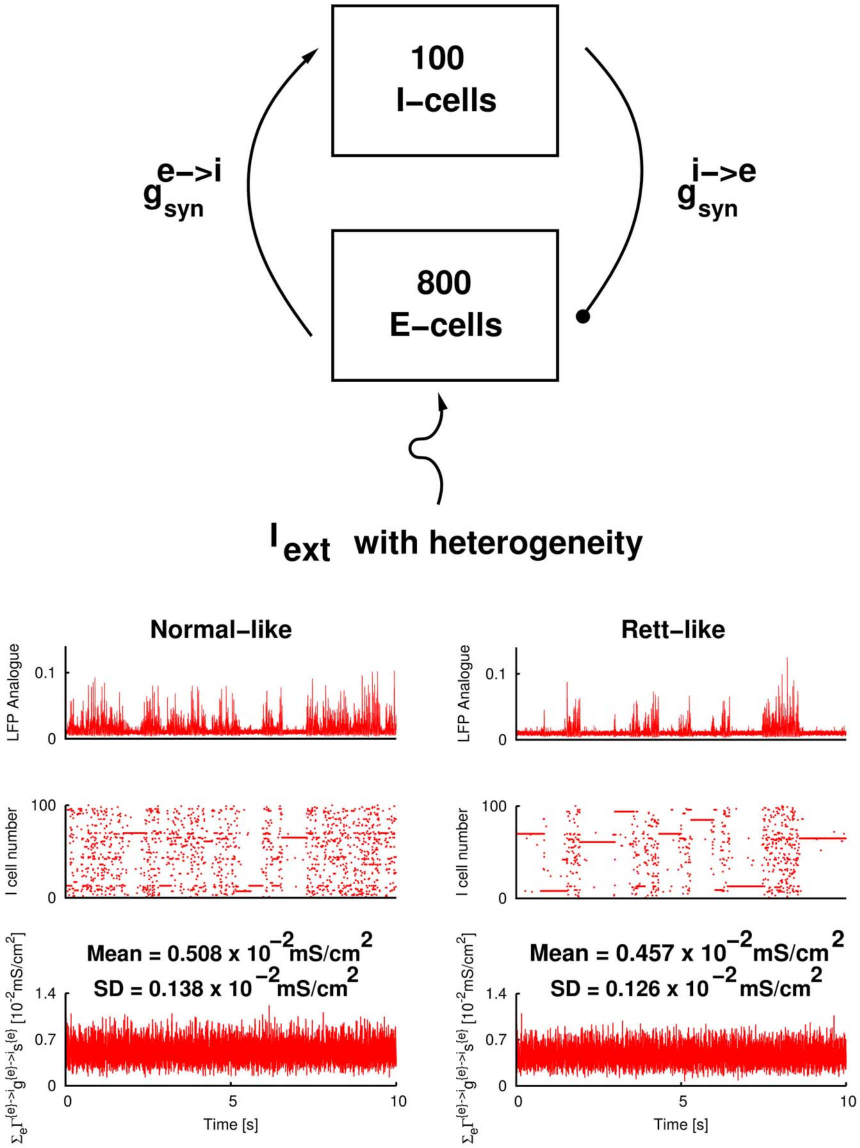

Figure 3. Simulated hippocampal slow population activities for normal and Rett when excitatory cells are explicitly included. Top Schematic of the model setup. **Bottom left** $g_{syn}^{e\to i}=0.001$. **Bottom right** $g_{syn}^{e\to i}=0.0009$.
doi:10.1371/journal.pone.0091148.g003

possible to explain these experimental observations with our model mechanisms.

Reduced excitatory fluctuations in hippocampal network model simulations produce population activities with characteristics similar to those in MeCP2-null mice

In this section, we show, via a simulation approach, that decreased excitatory fluctuations are sufficient to account for all the observations by [13] with regard to the different hippocampal SPA characteristics in MeCP2-null mice. In the next section, we address the apparent paradox of hyper-excitable responses from MeCP2-null samples as compared to the wild type ones.

In Figure 2 we show how varying the amount of excitatory fluctuations of model inhibitory interneuronal network can simulate SPA characteristics of both wild type and MeCP2-null samples. In the simulations of Figure 2, we artificially drive the 100 all-to-all coupled model inhibitory interneurons with fluctuating inputs as shown in the schematic, and as done previously in [22] (see sec:mm and equation set 4). All the parameters used for these simulations are identical, except that the g_{e0} (mean excitatory levels) and σ_e (excitatory fluctuations) are changed as shown (see figure caption and Table 3 for parameter values used). Four LFP example plots are shown, and for three of the examples, raster plots of firings of each of the 100 model inhibitory interneurons during the 10-second interval and the actual excitatory input conductance to a randomly selected model interneuron in the network (i.e. $\sum_{m;m\neq l;m\in pyr}\Gamma_{m\to l}\times g_{syn}^{m\to l}\times s_m$, for a particular neuron l–see equation set 3) is shown.

It is clear from the four LFP model examples (inside the box of Figure 2) that the simulations with the lower σ_e values (top two LFP plots in the box) have a lower SPA frequency than the ones with a higher σ_e values (bottom two). The top two model LFPs also appear to have larger magnitudes than the bottom ones. It is also clear that there is not much difference in SPA frequencies when only g_{e0} (but not σ_e) is changed (compare the top-left versus top-

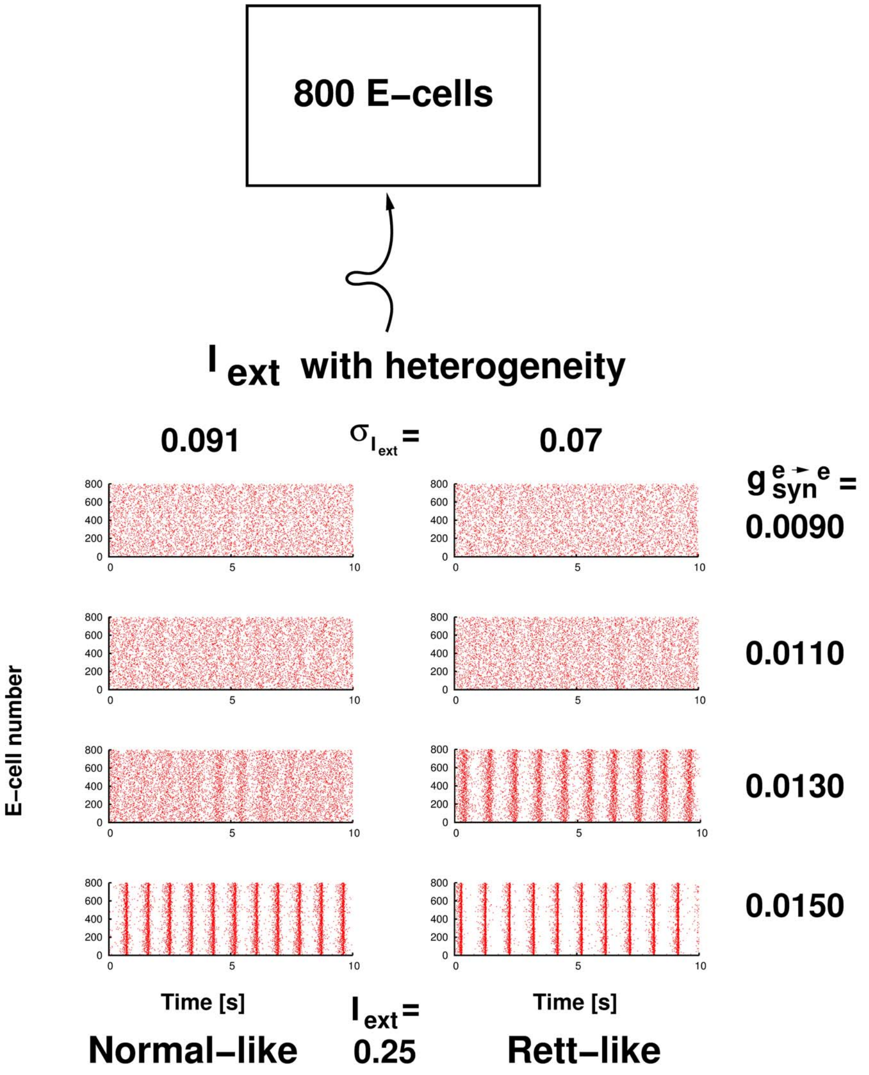

Figure 4. Network bursting of excitatory pyramidal cells affected by excitatory fluctuations.
doi:10.1371/journal.pone.0091148.g004

right LFP plots, and the bottom-left versus bottom-right LFP plots).

The excitatory input for the top-left LFP example (in box of Figure 2) has smaller fluctuations (i.e., a lower standard deviation) and also has a smaller mean value, as expected from the smaller g_{e0} and σ_e values used for the simulations. The calculated values of mean and SD (standard deviation) values of the 10-second trace are labelled on top of this panel. As expected, these values agree with the g_{e0} and σ_e values used as inputs. Given the experimental observations described above, we consider the top LFP network dynamics (the top two LFP plots in the box) to be "Rett-like" and the bottom ones to be "normal-like", and are so labelled in Figure 2.

The simulation results shown in Figure 2 are from inhibitory networks with virtual excitatory drive as schematized (top-right drawing of Figure 2). Rett-like and normal-like output states are seen to occur, as understood by our previously determined mechanistic understanding [22]. We wondered whether these states would continue to hold if excitatory networks were explicitly included. To this end, we developed inhibitory-excitatory networks (see sec:mm for model details). Figure 3 depicts the situation when excitatory cells are explicitly rather than virtually included, as schematized in the figure. The model inhibitory interneurons are driven by actual model pyramidal cells and not by artificial inputs as in Figure 2. We used the same I_{ext} distribution to drive the model pyramidal cells for normal- and Rett-like scenarios (see Table 3). To simulate the lower excitatory fluctuations received by inhibitory interneurons for MeCP2-null scenario, we use a lower $g_{syn}^{e\to i}$ value in Figure 3 ('Rett-like' in right column) than the 'normal-like' in the left column. The top row shows the simulated SPAs (in LFP representation). Similar to Figure 2, we also show inhibitory cell firings in raster plots (second row), and excitatory input conductance to a randomly selected model interneuron in the network (third row). However, in this case, it is an explicit excitatory input, rather than virtual excitatory input as in Figure 2. We calculate the mean and SD values of the 10-second trace and label the results on top of the panels. From these numbers it is obvious that the inhibitory population of the 'Rett-like' state receives lower excitatory input (both in mean and SD) as compared to the 'normal-like' state.

We conclude that since simulated hippocampal SPAs can still be generated when excitatory cells are explicitly included, SPAs as a fluctuation-driven phenomenon (as described by [22]) are robust and the gross characteristics of SPAs are mainly dependent on the

Table 2. Intrinsic parameters used for network simulations.

Parameter	Description	Units
V_{max}	see equation set 1	mV
V_{reset}		mV
I_{ext}	see equation set 2	$\mu A/cm^2$
V_{th}		mV
V_{peak}		mV
V_{rest}		mV
α		$(mS)(mV)^{-1}/cm^2$
λ		ms^{-1}
d		$\mu A/cm^2$
β		mS/cm^2
k		dimensionless

doi:10.1371/journal.pone.0091148.t002

amount of fluctuations, and less dependent on the particular way in which fluctuations are generated. We also note that in lowering the $g_{syn}^{e\to i}$, we decrease both the mean excitatory level and the excitatory fluctuations received by inhibitory interneurons in Figure 3 (right column) as compared to Figure 3 (left column). However, it is the decrease in excitatory fluctuations which is primarily responsible for the observed changes in SPA characteristics. This point is clearly illustrated in Figure 2 where we vary g_{e0} and σ_e separately, and we see the SPA characteristics being much more sensitive to changes in σ_e than to changes in g_{e0}. With the explicit representation of excitatory cell networks, it is not easily possible to separately change g_{e0} and σ_e. We chose not to do a more formal quantification of population frequency changes as done in [22] since the population frequency changes were obvious here.

Reduced excitatory fluctuations promote a hyper-excitable response in networks of model pyramidal cells

As already observed by [13] and others (e.g. [11]), there appears to be a decrease in excitatory activities in Rett mice. However, [13] found that when brief trains of high frequency stimulation (HFS), a protocol commonly used to induce long term potentiation (LTP), were applied to MeCP2-null hippocampal slices, sharp wave-like or excitatory network bursting activities were readily induced, whereas this was not the case in the wildtype slices. In other words, the MeCP2-null slices had a hyper-excitable response. Given the insight and predictions derived from our mathematical models, can we explain this seemingly paradoxical result?

Building on mechanistic understandings from our previous work [22], we showed in the previous section that reduced excitatory fluctuations could explain the changed characteristics of hippocampal SPAs produced in MeCP2-null slices relative to wildtype. In this section, we show via simulations, that this hyper-excitable response, as expressed by the induction of excitatory network bursting is sensitive to the amount of excitatory fluctuations. We first make the reasonable, albeit somewhat simplistic, assumption that the LTP induction protocol causes an increase in excitatory synaptic strengths, so that in our network simulations we examine the network output under these conditions. Next, as we show and describe in detail below, decreased excitatory fluctuations (as observed by [13] on MeCP2-null samples through decreased EPSC activities–see the distributions of EPSC charges in Figure 6B of their publication) can facilitate excitatory network bursting. This is achieved by allowing the transition from tonic firing to network bursting to occur at a lower excitatory-excitatory synaptic strength ($g_{syn}^{e\to e}$).

Figure 4 shows an example of our simulation results. As described in sec:mm and Table 3, each simulation in Figure 4 is an 800-cell excitatory network, as schematized at the top of Figure 4. Each pyramidal cell is driven by fluctuating input given by a constant I_{ext}, and fluctuations introduced via the magnitude of absolute heterogeneity of I_{ext} and a 5% relative heterogeneity introduced into the intrinsic properties of the pyramidal cells (see Table 3). Left columns are simulations in which a higher I_{ext} heterogeneity is used (SD of I_{ext} is $0.091\mu A/cm^2$), while the right columns are simulations that are under a lower I_{ext} heterogeneity (SD of I_{ext} is $0.07\mu A/cm^2$). The simulations are arranged side by side with the ones having a lower $g_{syn}^{e\to e}$ value on the top (the $g_{syn}^{e\to e}$ value used in the simulations are depicted on the right side of each pair of simulations).

It is clear from these simulations that the excitatory network transitions from a tonic-like mode (random, incoherent firing of pyramidal cells in the network) to a bursting-like mode (synchronized, coherent firing of pyramidal cells) as the excitatory-excitatory synaptic strength values ($g_{syn}^{e\to e}$) are increased (which we interpret to be due to an LTP induction of brief trains of HFS). However, comparison between the left column and the right column reveals that, other conditions being the same, given a lower absolute I_{ext} heterogeneity (which represents lower input excitatory fluctuations), the network transitions to a bursting-like state at a lower value of $g_{syn}^{e\to e}$. One can then interpret the apparent paradoxical HFS results of [13] in light of these simulation results as follows: assuming everything else identical, lower excitatory fluctuation levels in the MeCP2-null samples leads to a hyper-excitable response as given by facilitating the induction of network bursting via HFS, because the same increase in excitatory synaptic strength $g_{syn}^{e\to e}$ brings the MeCP2-null sample to a network bursting regime (lower $g_{syn}^{e\to e}$ threshold for transition) but not for the wild-type sample (higher $g_{syn}^{e\to e}$ threshold for transition). That is, note the second and third rows of the raster plots in Figure 4.

Table 3. Summary of parameters and synaptic connectivities used in the simulations.

	Intrinsic properties		Synaptic properties (equation set 3)	Driving force (format of I_{ext})
	Pyramidal cells	Interneurons		
Figure 2	Not applicable	$\alpha = 1$	$P(i \to i) = 1$	equation set 4:
		$\lambda = 0.81$	$g_{syn}^{i \to i} = 0.041$	$E_e = 0$
		$d = 4.0$	$E_i = -73$	$\tau_e = 3$
		$V_{rest} = -61$	$s_{max} = 0.8$	see figure captions for g_{e0} and σ_e values
		$V_{th} = -61$	$\tau_i = 10$	
		$\beta = 0$		
		$k = 0$		
		$V_{reset} = -65$		
		$V_{peak} = 0$		
		$V_{max} = 15$		
Figure 3	$\alpha = \begin{cases} 0.0006 & V < V_{th} \\ 0.02 & \text{otherwise} \end{cases}$	Parameters for interneuronal population same as Figure 2.	$P(i \to i) = 1$	Constant value for each pyramidal cell, with absolute Gaussian heterogeneity among pyramidal cell population (mean $I_{ext} = 1.45$, SD = 0.3). $I_{ext} = 0$ for interneurons.
	$\lambda = 0.005$		$P(i \to e) = 0.07$	
	$d = 0.8$		$P(e \to i) = 0.4$	
	$V_{rest} = -65$		$P(e \to e) = 0.07$	
	$\beta = -0.004$		$g_{syn}^{i \to i} = 0.041$	
	$k = 1$		$g_{syn}^{i \to e} = 0.0009$	
	$V_{th} = -18.33$		$g_{syn}^{e \to e} = 0.002$	
	$V_{peak} = 30$		$E_e = 0$	
	$V_{max} = 30$		$E_i = -73$	
	$V_{reset} = -55$		$s_{max} = 0.8$	
			$\tau_e = 3$	
			$\tau_i = 10$	
			see figure captions for $g_{syn}^{e \to i}$	
Figure 4	Parameters for pyramidal cell population same as Figure 3, except here we also introduce a 5% relative heterogeneity to λ and d values (uniform distributed central value $\pm 2.5\%$).	Not applicable	$P(e \to e) = 0.07$	Constant value for each pyramidal cell, with absolute Gaussian heterogeneity among pyramidal cell population (see figure caption for specific mean and SD values).
			$E_e = 0$	
			$\tau_e = 3$	
			$s_{max} = 0.8$	

doi:10.1371/journal.pone.0091148.t003

Discussion

Summary of results

An essential aspect in understanding Rett syndrome is to study how defects at the genetic level manifest at the cellular and network levels, leading to behavioural abnormalities. Performing experiments is one way we can explore how cellular and network behaviours are subtly affected by genetic defects. However, owing to the complex relationship between variables and processes at different levels, it can be difficult to elucidate disease mechanisms by experiments alone. Mathematical modelling and simulations can help experimenters by pinpointing variables that are likely to have causal relationships with experimental results. In this study, we have used mathematical modelling and simulations to paint a picture of how hippocampal slow population activities (SPAs) are affected in a mouse model of Rett syndrome. Three principle observations emerge from our work: 1) changes in mean excitatory drive within the hippocampal network are not necessarily a critical component of these network alterations, 2) decreased excitatory fluctuations can be a critical factor in changes in hippocampal network oscillations in Rett syndrome, and 3) these fluctuations in excitatory levels can also explain hyper-excitable responses in MeCP2-deficient hippocampal networks. For the first time, we have brought together observations, some of them seemingly paradoxical (decreasing basal excitation, increasing susceptibility to hyper-excitable responses with high frequency stimulation, and changes in characteristics of slow population activities in MeCP2-null slices), under the unifying framework of excitatory fluctua-

tions. We found that decreasing excitatory fluctuations is sufficient to account for all of the observations in [13]. Our work highlights the importance of the often overlooked role of excitatory fluctuations in shaping biological and pathological hippocampal population activities [37,38]. Furthermore, the results illustrate how mathematical modelling can be used to gain insight into novel mechanisms involved in the pathogenesis of disease states and facilitate the targeting of novel therapeutic strategies.

Excitatory fluctuations and network hyper-excitable responses

Our model results indicating that oscillatory activity could be dramatically influenced by fluctuations led to the question of whether the fluctuation component could also explain hyper-excitable responses seen in MeCP2-deficient hippocampal networks. For this to be examined, we took advantage of previous excitatory network models expressing population bursts [32], and varied excitatory fluctuations. Intriguingly, we found that networks with decreased excitatory fluctuations could also be hyper-excitable, allowing excitatory, sharp wave-like bursting patterns to emerge for smaller excitatory coupling strengths. This is consistent with the hyper-excitable response seen in MeCP2-deficient hippocampal networks, as population bursting events were readily induced by an excitatory stimulus that had no effect on wild-type hippocampal networks. As such, our mathematical modelling results implicate the decreased excitatory fluctuation component as the essential driving force for both reduced network oscillatory (i.e., slower frequency population activities) and also hyper-excitable responses.

While it might seem paradoxical that reduced excitatory fluctuations can lead to network hyper-excitable responses (Figure 4), our simulation results should be viewed in the context of the overall excitation experienced by the pyramidal cell population. To determine why reduced excitatory fluctuations can increase the propensity of the network to burst, we ran several additional sets of simulations in the spirit of Figure 4 but with different magnitudes of I_{ext} (while keeping the SDs of I_{ext} the same as shown in Figure 4). We discovered that our results held if I_{ext} was close to the firing threshold of individual model pyramidal cells (the threshold of the model pyramidal cells is $\sim 0.24 \mu A/cm^2$, thus the I_{ext} in Figure 4 is slightly above threshold). If I_{ext} is too far below the threshold, the reverse result happens, that is, the network with larger excitatory fluctuations bursts more easily. The transition from tonic-like to bursting occurs for smaller excitatory connection strengths relative to the network with smaller excitatory fluctuations. This is understandable since when I_{ext} is well below threshold, the entire network is mainly driven by fluctuations [39]. Therefore, larger fluctuations are needed to make the network excitable in the first place, allowing neurons to cross the threshold and fire. However, in the regime where I_{ext} is slightly below or above threshold, the network relies less on fluctuations to drive the cells. Instead, increasing fluctuations disrupts the synchronization of pyramidal cells, making the network with larger excitatory fluctuations less burst-prone.

Model Interpretations and Limitations

Ideally, one would like to have a single model network which incorporates both excitatory and inhibitory populations, and be able to recapitulate all the aspects of neuronal activities in MeCP2-null slice samples. We did not pursue this approach in this work, however. Instead, we teased apart the network into inhibitory (Figure 2), excitatory-inhibitory (Figure 3) and excitatory (Figure 4) components to explore different features of MeCP2-null networks. This is somewhat justified because of our reliance on model mechanistic understandings [22,32] in this work. Further, we note that the number of neurons in our models is a drastic reduction of the total number of neurons in actual hippocampal slices, and a mismatch between the excitation needed to drive the hippocampal population activities (Figure 3) and sharp wave-like bursts (Figure 4) would occur. In Figure 3, we had to make the model pyramidal cells fire at a relatively high frequency (to make up for the small number of model excitatory cells) to provide enough drive to obtain hippocampal population activities. However, the same amount of injected current I_{ext} to the model pyramidal cells in Figure 4 would have resulted in too high a sharp wave-like bursting frequency. One can envision that with the number of model neurons appropriately reflecting actual biological networks, each model pyramidal cell can be less excitable and still be able to support hippocampal population activities as in Figure 3. One would then in principle be able to exploit the full-sized model to infer the possible mean excitation of the network, given the experimental network bursting data. Our current models are not able to pinpoint actual mean excitation levels, other than to conclude that the mean excitation needs to be in the vicinity of the threshold of individual pyramidal cells for our simulations (Figure 4) to be reflective of the biological situation. Furthermore, we note that the excitatory sharp waves seen in the experiment are interpreted to be due to synchronized firing of excitatory cells as shown in the raster plots of Figure 4 as network bursts. Although we cannot directly assess whether the model mechanism underlying the network bursts are similar to the sharp waves observed in experiment, it is reasonable to assume that synchronized excitatory firings contribute to the sharp waves seen in experiment. The difference in population frequency between model and experiment is likely due to the smaller number of neurons in the model compared to experiment, as mentioned above. More critically, using only excitatory networks to explain the hyper-excitable response neglects feedback inhibition. However, in the absence of more realistic network sizes and configurations, we felt it premature to directly consider this here. This will be developed in future models.

In the network models employed, two features are represented: excitatory drive (g_{e0}) and excitatory fluctuations (σ_e). In these models, these features could be represented separately. The excitatory drive component reflects the mean level of excitatory conductance present in the model network. The excitatory fluctuation is represented generically, and is defined as the standard deviation of the excitatory conductance (see sec:mm) and represents the firing of many pre-synaptic neurons and thousands of stochastically-releasing synapses [40,41]. However, in the experimental, hippocampal slice preparation, the mean excitatory component and excitatory fluctuation component are not necessarily distinct. The mean excitatory drive represents a combination of the frequency and amplitude of excitatory currents that sets a tonic level of excitation. The excitatory fluctuation reflects the spatial and temporal degree of basal EPSC activity, and the range associated with individual EPSC amplitudes. There are known morphological changes in Rett syndrome, such as in dendritic spine densities, which could relate to excitatory fluctuation changes [42–44], but it is unclear at present whether this translates to other brain regions.

Concluding Remarks

In conclusion, the work presented here indicates that decreased excitatory fluctuations, and not just the level of excitatory drive, can be instrumental in ascribing the functional properties of a network system. For Rett syndrome, where network hyper-

excitable responses co-exist with attenuated spontaneous excitatory drive, our network models allowed us to dissect out how such seemingly paradoxical states could co-exist. Our results surprisingly show that attenuated excitatory fluctuations can in fact lead to a robust decrease in inhibitory population activities and simultaneously allow for excitatory network hyper-excitable responses to manifest. This has clear therapeutic implications for MeCP2-deficient systems, as it suggests that an increase in excitatory fluctuations, and not necessarily changes in tonic excitatory levels, would restore the normal dynamics of the network and decrease hyper-excitable responses.

Acknowledgments

The authors would like to thank the reviewers for their constructive comments.

Author Contributions

Conceived and designed the experiments: ECYH JHE LZ FKS. Performed the experiments: ECYH LZ. Analyzed the data: ECYH. Contributed reagents/materials/analysis tools: ECYH JHE LZ FKS. Wrote the paper: ECYH JHE LZ FKS.

References

1. Amir R, Van den Veyver I, Wan M, Tran C, Francke U, et al. (1999) Rett syndrome is caused 453 by mutations in x-linked MeCP2, encoding methyl-CpG-binding protein 2. Nat Genet 23(2): 185–188.
2. Moretti P, Zoghbi H (2006) MeCP2 dysfunction in Rett syndrome and related disorders. Curr Opin Genet Dev 16(3): 276–281.
3. Lane J, Lee H, Smith L, Cheng P, Percy A, et al. (2011) Clinical severity and quality of life in children and adolescents with Rett syndrome. Neurology 77(20): 1812–1818.
4. Neul J (2012) The relationship of Rett syndrome and MeCP2 disorders to autism. Dialogues Clin Neurosci 14(3): 253–262.
5. Johnston M, Mullaney B, Blue M (2003) Neurobiology of Rett syndrome. J Child Neurol 18(10): 688–692.
6. Nomura Y, Segawa M (2005) Natural history of Rett syndrome. J Child Neurol 20(9): 764–768.
7. Stauder JEA, Smeets EEJ, van Mil SGM, Curfs LGM (2006) The development of visual- and auditory processing in Rett syndrome: an ERP study. Brain Dev 28(8): 487–494.
8. Glaze D (2005) Neurophysiology of Rett syndrome. J Child Neurol 20(9): 740–746.
9. JourdanMoser S,Weber P, Lütschg J (2007) Rett syndrome: clinical and electrophysiologic aspects. Pediatr Neurol 36(2): 95–100.
10. Katz DM, Berger-Sweeney JE, Eubanks JH, Justice MJ, Neul JL, et al. (2012) Preclinical research in Rett syndrome: setting the foundation for translational success. Dis Model Mech 5(6): 722–745.
11. Dani V, Chang Q, Maffei A, Turrigiano GG, Jaenisch R, et al. (2005) Reduced cortical activity due to a shift in the balance between excitation and inhibition in a mouse model of rett syndrome. PNAS 102: 12560–12565.
12. Dani VS, Nelson SB (2009) Intact long-term potentiation but reduced connectivity between neo cortical layer 5 pyramidal neurons in a mouse model of Rett syndrome. J Neurosci 29(36): 11263–11270.
13. Zhang L, He J, Jugloff DG, Eubanks JH (2008) The MeCP2-null mouse hippocampus displays altered basal inhibitory rhythms and is prone to hyperexcitability. Hippocampus 18(3): 294–309.
14. Wood L, Gray NW, Zhou Z, Shepherd GM (2009) Synaptic circuit abnormalities of motor-frontal layer 2/3 pyramidal neurons in an RNA interference model of methyl-CpG-binding protein 2 deficiency. J Neurosci 29(40): 12440–12448.
15. Wood L, Shepherd GM (2010) Synaptic circuit abnormalities of motor-frontal layer 2/3 pyramidal neurons in a mutant mouse model of Rett syndrome. Neurobiol Dis 38(2): 281–287.
16. Stettner GM, Huppke P, Brendel C, Richter DW, Gärtner J, et al. (2007) Breathing dysfunctions associated with impaired control of postinspiratory activity in MeCP2-/y knockout mice. J Physiol 579: 863–876.
17. Medrihan L, Tantalaki E, Aramuni G, Sargsyan V, Dudanova I, et al. (2008) Early defects of GABAergic synapses in the brain stem of a MeCP2 mouse model of Rett syndrome. J Neurophysiol 99(1): 112–121.
18. Taneja P, Ogier M, Brooks-Harris G, Schmid DA, Katz DM, et al. (2009) Pathophysiology of locus ceruleus neurons in a mouse model of Rett syndrome. J Neurosci 29(39): 12187–12195.
19. Abdala AP, Dutschmann M, Bissonnette JM, Paton JF (2010) Correction of respiratory disorders in a mouse model of Rett syndrome. PNAS 107(42): 18208–18213.
20. Kline DD, Ogier M, Kunze DL, Katz DM (2010) Exogenous brain-derived neurotrophic factor rescues synaptic dysfunction in MeCP2-null mice. J Neurosci 30(15): 5303–5310.
21. Wu CP, Luk WP, Gillis J, Skinner F, Zhang L (2005) Size does matter: Generation of intrinsic network rhythm in thick mouse hippocampal slices. J Neurophysiol 93: 2302–2317.
22. Ho ECY, Strüber M, Bartos M, Zhang L, Skinner FK (2012) Inhibitory networks of fast-spiking interneurons generate slow population activities due to excitatory fluctuations and network multi stability. J Neurosci 32(29): 9931–9946.
23. Calfa G, Hablitz J, Pozzo-Miller L (2011) Network hyperexcitability in hippocampal slices from MeCP2 mutant mice revealed by voltage-sensitive dye imaging. J Neurophysiol 105(4): 1768–1784.
24. McLeod F, Ganley R, Williams L, Selfridge J, Bird A, et al. (2013) Reduced seizure threshold and altered network oscillatory properties in a mouse model of Rett syndrome. Neuroscience 231: 195–205.
25. Guy J, Gan J, Selfridge J, Cobb S, Bird A (2007) Reversal of neurological defects in a mouse model of Rett syndrome. Science 315(5815): 1143–1147.
26. Asaka Y, Jugloff D, Zhang L, Eubanks J, Fitzsimonds R (2006) Hippocampal synaptic plasticity is impaired in the MeCP2-null mouse model of Rett syndrome. Neurobiol Dis 21(1): 217–227.
27. Jugloff DG, Logan R, Eubanks JH (2006) Breeding and maintenance of an MeCP2-deficient mouse model of rett syndrome. J Neurosci Meth 154(1–2): 89–95.
28. Wu CP, Nassiri Asl M, Gillis J, Skinner FK, Zhang L (2005) An in vitro model of hippocampal sharp waves: Regional initiation and intracellular correlates. J Neurophysiol 94: 741–753.
29. Wu C, Shen H, Luk WP, Zhang L (2002) A fundamental oscillatory state of isolated rodent hip pocampus. J Physiol 540: 509–527.
30. Ho ECY, Zhang L, Skinner FK (2009) Inhibition dominates in shaping spontaneous CA3 hip pocampal network activities. Hippocampus 19: 152–165.
31. Izhikevich EM (2003) Simple model of spiking neurons. IEEE Trans Neural Networks 14: 1569–1572.
32. Dur-e-Ahmad M, Nicola W, Campbell SA, Skinner FK (2012) Network bursting using experimentally constrained single compartment CA3 hippocampal neuron models with adaptation. J Comput Neurosci 33(1): 21–40.
33. Ho ECY (2011) If you want to be slow you have to be fast: Control of slow population activities by fast-spiking interneurons via network multistability. Ph.D. thesis, University of Toronto.
34. Rudolph M, Piwkowska Z, Badoual M, Bal T, Destexhe A (2004) A method to estimate synaptic conductances from membrane potential fluctuations. J Neurophysiol 91(6): 2884–2896.
35. Taxidis J, Coombes S, Mason R, Owen MR (2012) Modeling sharp wave-ripple complexes through a CA3-CA1 network model with chemical synapses. Hippocampus 22(5): 995–1017.
36. Loken C, Gruner D, Groer L, Peltier R, Bunn N, et al. (2010) SciNet: Lessons learned from building a power-efficient top-20 system and data centre. J Phys: Conf Ser 256: 012026.
37. de la Prida LM, Huberfeld G, Cohen I, Miles R (2006) Threshold behavior in the initiation of hippocampal population bursts. Neuron 49: 131–142.
38. Takano H, McCartney M, Ortinski PI, Yue C, Putt ME, et al. (2012) Deterministic and stochastic neuronal contributions to distinct synchronous CA3 network bursts. J Neurosci 32: 4743–4754.
39. Schreiber S, Samengo I, Herz AVM (2009) Two distinct mechanisms shape the reliability of neural responses. J Neurophysiol 101: 2239–2251.
40. Destexhe A, Rudolph M, Fellous JM, Sejnowski TJ (2001) Fluctuating synaptic conductances recreate in vivo-like activity in neocortical neurons. Neuroscience 107: 13–24.
41. Ribrault C, Sekimoto K, Triller A (2011) From the stochasticity of molecular processes to the variability of synaptic transmission. Nat Rev Neurosci 12(7): 375–387.
42. Belichenko PV, Wright EE, Belichenko NP, Masliah E, Li HH, et al. (2009) Widespread changes in dendritic and axonal morphology in MeCP2-mutant mouse models of Rett syndrome: evidence for disruption of neuronal networks. J Comp Neurol 514(3): 240–258.
43. Chapleau CA, Calfa GD, Lane MC, Albertson AJ, Larimore JL, et al. (2009) Dendritic spine pathologies in hippocampal pyramidal neurons from rett syndrome brain and after expression of Rett-associated MeCP2 mutations. Neurobiol Dis 35(2): 219–233.
44. Chapleau CA, Boggio EM, Calfa G, Percy AK, Giustetto M, et al. (2012) Hippocampal CA1 pyra-midal neurons of MeCP2 mutant mice show a dendritic spine phenotype only in the presymptomatic stage. Neural Plast Epub: 2012:976164.
